# Evaluating the implementation of a personal health record for chronic primary and secondary care: a mixed methods approach

**DOI:** 10.1186/s12911-019-0969-7

**Published:** 2019-11-27

**Authors:** Floor Sieverink, Saskia Kelders, Annemarie Braakman-Jansen, Julia van Gemert-Pijnen

**Affiliations:** 1 Centre for eHealth and Wellbeing Research, Department of Psychology, Health and Technology, University of Twente, P.O. Box 217, 7500 AE Enschede, the Netherlands; 2 Optentia Research Focus Area, North-West University, Vanderbijlpark, South Africa

**Keywords:** Implementation, Personal health record, T2DM, CHF, COPD, Mixed-methods

## Abstract

**Background:**

Personal health records (PHRs) provide the opportunity for self-management support, enhancing communication between patients and caregivers, and maintaining and/or improving the quality of chronic disease management. Their implementation is a multi-level and complex process, requiring a holistic approach that takes into account the technology, its users and the context of implementation. The aim of this research is to evaluate the fidelity of a PHR in chronic care (the degree to which it was implemented as intended) in order to explain the found effects.

**Methods:**

A convergent parallel mixed methods design was used, where qualitative and quantitative data were collected in parallel, analyzed separately, and finally merged. Log data of 536 users were used to gain insight into the actual long-term use of the PHR (the dose). Focus group meetings among caregivers (*n* = 13) were conducted to assess program differentiation (or intended use). Interviews with caregivers (*n* = 28) and usability tests with potential end-users (*n* = 13) of the PHR were used to understand the responsiveness and the differences and similarities between the intended and actual use of the PHR**.**

**Results:**

The results of the focus groups showed that services for coaching are strongly associated with monitoring health values and education. However, the PHR was not used that way during the study period. In the interviews, caregivers indicated that they were ignorant on how to deploy the PHR in current working routines. Therefore, they find it difficult to motivate their patients in using the PHR. Participants in the usability study indicate that they would value a PHR in the future, given that the usability will be improved and that the caregivers will use it in daily practice as well.

**Conclusions:**

In this study, actual use of the PHRs by patients was influenced by the responsiveness of caregivers. This responsiveness is likely to be strongly influenced by the perceived support when defining the differentiation and delivery of the PHR. A mixed-methods approach to understand intervention fidelity was of added value in providing explanations for the found effects that could not be revealed by solely focusing on the effectiveness of the technology in an experimental trial.

## Background

Following the most recent numbers, approximately 11% of the Dutch population is registered as having type 2 diabetes mellitus (T2DM), congestive heart failure (CHF) and/or COPD, and it is expected that these numbers will only grow in the upcoming years [1–3]. With this growing prevalence, a number of issues arise. For example, care providers are responsible for a growing number of tasks concerning the treatment and counseling of chronically ill people. At the same time, the healthcare system largely focuses on acute illnesses, while ageing with a chronic disease is becoming the norm [4]. Sustainable solutions are therefore needed to realize a transformation in health care delivery and to support the shifts from 1) institutionalized care to home care; 2) acute episodic care to more continuous chronic care; and 3) the patient as a passive recipient of care to an active patient that is able to self-manage [5].

eHealth, and more specifically Personal Health Records (PHRs), provide the opportunity for self-management support and maintaining and/or improving the quality of chronic disease management by engaging patients in their own healthcare [6–8]. Originally, PHRs were defined as applications that allow individuals to access, manage, and share health information with their (informal) caregivers [6]. However, to improve chronic health care, to enhance communication between patients and caregivers, and to support patients in developing self-management skills, a PHR must be more than just a repository for managing and sharing health information [6]. Therefore, additional functions for supporting patients when working on health-related goals, learning more about (living with) chronic conditions and/or by supporting patient-provider communication provide the opportunity to support patients in taking control of their own health [7].

Despite the potential benefits of PHRs in chronic disease management, evidence regarding the effectiveness on outcomes such as improved self-management skills, clinical outcomes, or the organization of care remains sparse [9, 10] and inconclusive [11–15]. This is often due to difficulties in the implementation [16]. Many evaluations focus on the effectiveness of PHRs as stand-alone, patient-centered technologies. However, according to the definition by Eysenbach [17], eHealth is much more than just a thing or a tool: it is an infrastructure for knowledge dissemination, communication, and the organization of care. Furthermore, the view of caregivers, whose endorsement and engagement are seen as key for obtaining positive outcomes [18], is often missing in current evaluations. This implies that developing and implementing sustainable eHealth solutions is a multi-level and complex process [16, 19] that requires a holistic evaluation approach taking into account the relationship between intervention fidelity (the degree to which an intervention was implemented as intended [20]) and outcomes (the extent to which the intervention has reached its goals). Current literature states that five elements should be measured to gain insight into this relationship, namely: adherence (was the intervention used as intended [21]); exposure or dose (the amount of an intervention received by the users); quality of delivery (the manner in which a caregiver delivers a program), participant responsiveness (the extent to which users are engaged by the technology); and program differentiation (identifying the essential elements that are needed to reach the intended effects) [20]. The evaluation of these elements requires input from the perspectives of different stakeholders in different contexts, implying that the uptake of PHRs into routine care cannot be evaluated by focusing on only one level (e.g., the point of view of care providers) or outcome (e.g., glycemic control). However, up to now this holistic evaluation approach is often missing in PHR research. In this study, we therefore focus on a mixed methods approach to evaluate the fidelity of a PHR for patients with type 2 diabetes mellitus (T2DM), congestive heart failure (CHF), or COPD and how it influenced the obtained outcomes. The research questions are as follows:

1. How did the responsiveness of care providers regarding the PHR influence the actual use (exposure or dose) by patients?
2. To what extent does this dose match with the differentiation of the PHR as defined by caregivers (adherence)?
3. How can any differences between the intended and actual use (or adherence) be understood?

## Methods

To gain a more complete understanding of what difference the PHR can make in the context of self-management support for the patients and the working routines of caregivers and why, a mixed-methods evaluation approach will be applied combining quantitative and qualitative data on multiple levels [22].

### The e-Vita project

The PHR e-Vita was developed by the former foundation ‘Care Within Reach’, a partnership between Philips and Achmea (a Dutch health insurance company), with the goal to improve the quality of life of chronically ill people and to improve the accessibility to care facilities [23]. Two PHRs were developed for patients with T2DM and CHF. Based on these, a second version of the PHR was developed for patients with COPD.

The PHRs were implemented in primary care (T2DM and COPD) and secondary care (CHF) in the Netherlands for the duration of four trials to evaluate the effects of the PHR on the quality of life and health outcomes of patients with T2DM [24], CHF [25], and COPD [26]. The T2DM study included a randomized controlled trial to evaluate the effectiveness of a coaching service for self-management support [27]. A fifth research project was conducted to evaluate the impact of the PHR on health care utilization (cost-effectiveness). The evaluations of all three PHRs so far showed that the actual use of the PHR is lagging [28–30] and only marginal (health) improvements were found [29–32]. Concrete reasons for the underperformance of the PHR remained unclear in these studies.

The data described in this article are additionally collected in the context of a sixth study, a process evaluation that incorporated all three PHRs to understand their fidelity: the differences they can make in health care, why they can do so and why they may or may not have the expected impact.

### System and content of the PHR

The underlying system of the three PHRs was similar to a large extent, while the interfaces of the T2DM and CHF PHRs differed from the COPD PHR. Furthermore, the content differed depending on the condition. The PHR for CHF patients was linked to a system for telemonitoring. Patients were instructed to monitor their health values via that system on a daily basis, values were then transferred to the PHR. For the analyses in this study, we focus on the key features of the three PHRs as presented in Table 1.

**Table 1:** An overview of the key features of the PHRs

Feature	Explanation
Insight in health-related measurements	Users of the PHR for T2DM were able to see an overview (both textually and graphically) of a selection of T2DM-related health measurements from the past years, as performed at the annual check-up. A short explanation of each item was provided to support the participants with the interpretation of the values.
Education	All three PHRs contained disease- and lifestyle-related education.
Coaching	The PHRs for T2DM and COPD offer the users the possibility to add health-related goals, action plans and evaluations of these action plans. The PHR for CHF did not contain this function. The coaching function of the T2DM PHR was based on existing theories for behavior change, literature research and previous experiences and described elsewhere [25] .
Monitoring	CHF patients received equipment to monitor their weight, blood pressure, and heart rate on a daily basis (or individually adjusted in concordance with the heart failure nurse). When the monitored values were outside the pre-determined range or when no measurements were recorded, HF nurses received an alert and could subsequently contact the participant to possibly adjust the (medical) treatment. Patients with T2DM could track their health values using their own equipment (e.g., weight, blood pressure, waist circumference). Users with COPD could track their complaints and receive an advice on whether or not to consult their care provider. Furthermore, COPD users could regularly complete the Clinical COPD Questionnaire (CCQ) [31] to gain insight into the development of their clinical complaints.
Medication and co-morbidities	Users with CHF were asked to complete a list of medication and co-morbidities and to keep this list up-to-date. For the HF nurses, this overview could serve as an extra tool for the interpretation of deviating measurements.
Messaging	The COPD PHR contained an overview of caregivers with a service for sending and receiving messages to the caregivers.

### Mixed methods study

In the current study, a convergent parallel mixed methods design was used, where qualitative and quantitative data were collected in parallel, analyzed separately, and finally merged to gain a more complete understanding of how the PHR was delivered in the context of self-management support for the patients and the working routines of caregivers. An overview of the used data sources is given in Table 2.

**Table 2:** An overview of the used quantitative (Quan) and qualitative (Qual) data sources

Data	Quan/Qual	Goal
Log data	Quan	Gaining insight into how the PHRs were actually used on the long term by patients with T2DM, CHF, or COPD (exposure or dose)
Focus groups	Qual	Assessing how T2DM caregivers believe that the use of a PHR by patients adds value to their working routines (the intended use or program differentiation)
Interviews	Qual	Understanding the differences and similarities between the intended and actual use of the PHRs from the caregiver perspective (quality of delivery)
Usability tests	Qual	Understanding the differences and similarities between the intended and actual use of the PHRs from the patient perspective (potential responsiveness)

#### Log data

Because all features of the PHR were intended to be used frequently, a log data research protocol was used to gain insight into the actual long-term use of the PHRs for T2DM, CHF, and COPD throughout the study period [33]. The log data consisted of anonymous records containing information of every action by every user. For every action, the following information was collected: 1) An anonymous identification of the user; 2) the time and date of the action; 3) an identification of the action, and 4) optional additional information (e.g., what information was viewed by the users or which goal was added). All data was stored and processed following the current privacy regulations. The log data of the T2DM PHR was collected from 23 July 2013 to 4 January 2016; the CHF PHR log data was collected from 9 October 2013 to 25 December 2015; and the COPD PHR log data was collected from 30 June 2014 to 29 May 2016.

For every user, sessions (the actions taken between logging in and logging out) were identified first. All actions that were performed within half an hour after the last action, were considered to be part of the same session. Next, only the actions that indicated visiting or using one of the PHR features (e.g., adding a health value, adding a goal or action plan, opening an education topic) were selected for the analysis. To gain insight into the actual long-term use of the PHR features, the frequencies of visiting and actually using the features were calculated for every session and plotted in bar charts. The number of sessions on the X-axis was based on the number of sessions providing relevant information regarding the actual use of the features.

#### Focus groups

Because the unique and essential features and their dose (or program differentiation [20]) were not identified at the start of the implementation process, focus groups were conducted to gain insight into how T2DM caregivers (11 diabetes nurses and 2 dieticians) believe that the different features of the PHR can add value to their working routines in order to be consistent with national standards for T2DM care. All participants were involved in the daily care for T2DM patients in primary care, but were not caregivers in the e-Vita T2DM trial.

During these focus groups, all participants were first asked to describe and concretize their current tasks and activities within the different phases of T2DM treatment, based on the national guidelines [34]. Second, all participants received a short explanation and examples of the possibilities of monitoring, coaching, education, and logistic support via a PHR, and were then asked to discuss discuss how these features could be supportive in a reduction of their workload and the stimulation of self-management skills among their patients until consensus was reached. The duration of the focus groups was 2 h. Sound recordings were made and transcribed verbatim. The needs and wishes of the participants regarding the use of the potential key features of PHRs (monitoring, coaching, education, and logistic support) resulted into a new care pathway for T2DM that includes the deployment of a PHR.

#### Interviews and usability tests

To gain insight into quality of delivery of the PHRs and the potential responsiveness, the implementation process of the PHR, and to find explanations for any differences between the intended use (as defined in the focus groups) and actual use (as derived from the log data analyses), the results from 28 semi-structured in-depth interviews among T2DM (*n* = 11), CHF (*n* = 9), and COPD care providers (*n* = 8) who participated in the e-Vita trials were used. The interview scheme contained questions regarding the use of the PHR so far, the encountered barriers and facilitators in the actual implementation process, the results so far, and the potential changes the PHR can make in current working routines. The duration of the interviews was 45–60 min. A more extensive description of the interviews among the T2DM caregivers is provided elsewhere [35]. For this study, the transcripts from this interview study were used and combined with interviews among other caregivers to answer the current research questions.

To learn more about the responsiveness of patients when using the PHRs, 13 usability tests were conducted: 3 for the T2DM PHR and 10 for the COPD PHR. Since the functionality of the CHF PHR was limited, no usability tests were performed for this version. Due to privacy issues it was not possible to invite the actual patient users. Therefore, usability tests were conducted among potential end-users who fitted within the profile of the actual users (based on age and/or chronic condition). All usability tests (duration: 45–60 min) took place in the home environment of the participants. A think-aloud protocol with scenarios [36] was used to test all services of the PHRs (Table 1). An example of a scenario was: *“Your caregiver has advised you to lose weight. You set the goal to lose 10 kilograms. How would you add this goal to the PHR?”.* Where possible, the scenarios were kept the same across the different PHRs. Afterwards, participants were asked about their thoughts and feelings regarding the PHR.

All interviews with the care providers and usability tests with potential end-users were transcribed verbatim. Themes and categories were coded via open coding, axial coding and selective coding. In this way, recurring themes and items of interest that provide insight into the intervention fidelity were identified.

## Results

### Log data analysis

**Fig. 1:**
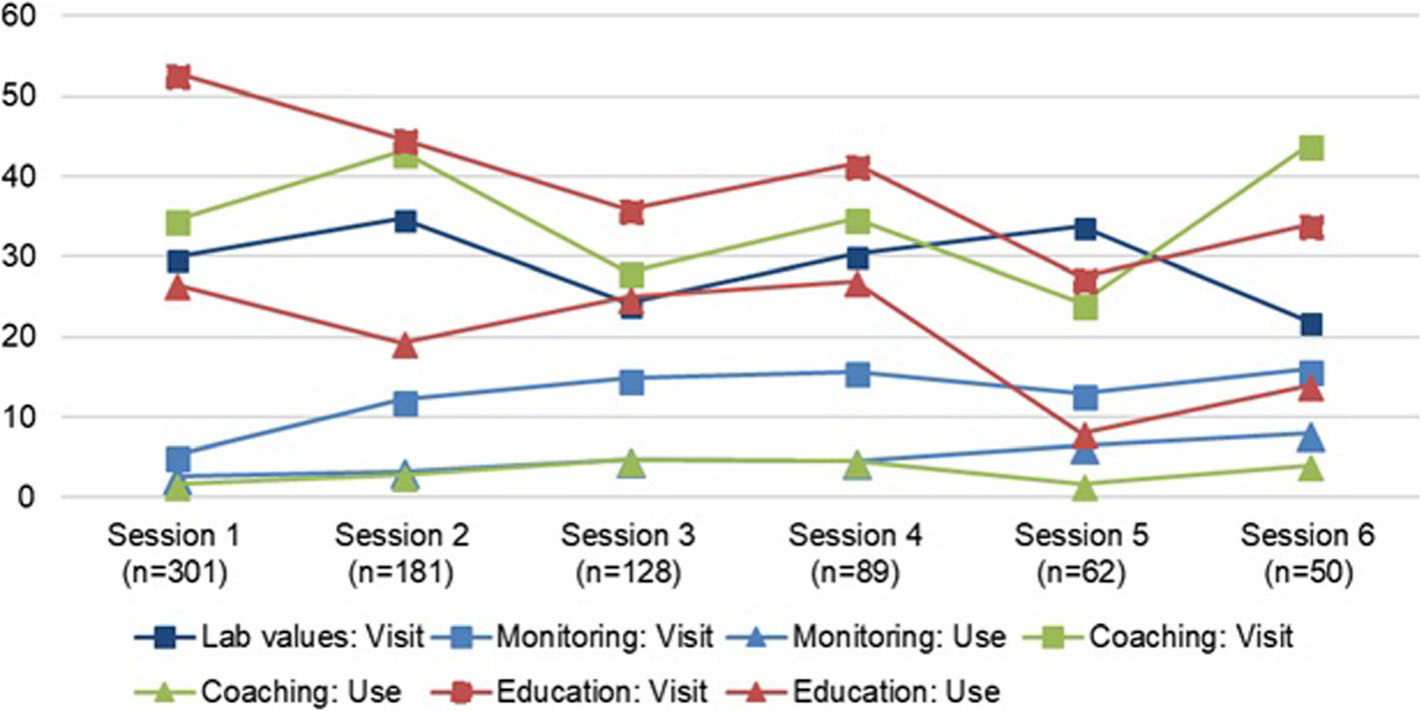
Usage per session and feature (T2DM). Monitoring action – Adding a new measurement, opening the overview of target values and monitoring history. Coaching action: adding a wish, goal, action or evaluation. Education action: opening education topic. No specific actions could be specified for lab values. % users = (number of different users visiting or using the feature in that session / total number of users in that session) * 100

**Fig. 2:**
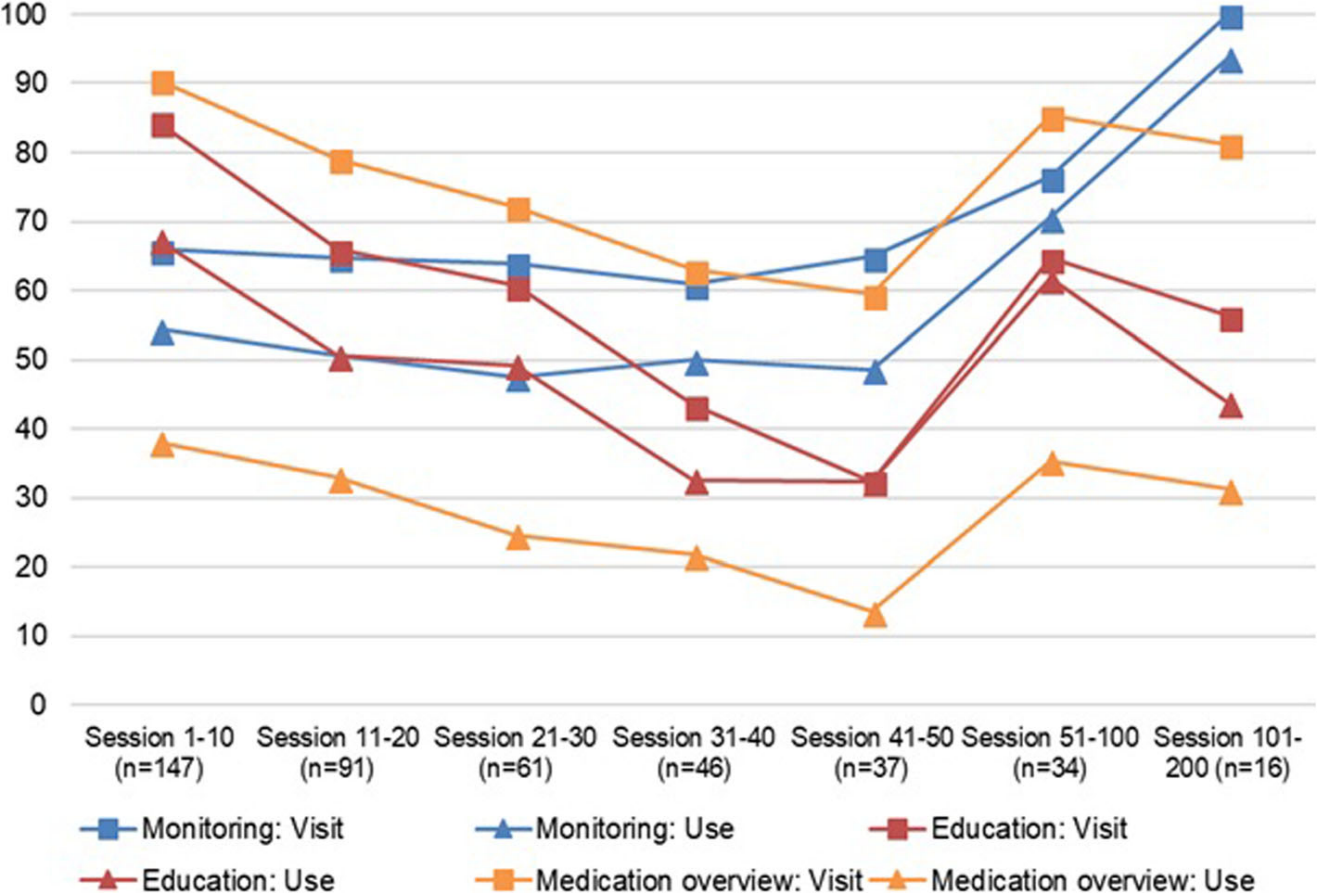
Usage per session and feature (CHF). Monitoring action: Adding a new measurement, opening the overview of target values and monitoring history; Education action: redirecting to external education website; Medication action: adding a medication. % users = (number of different users visiting or using the feature in that session / total number of users in that session) * 100

**Fig. 3:**
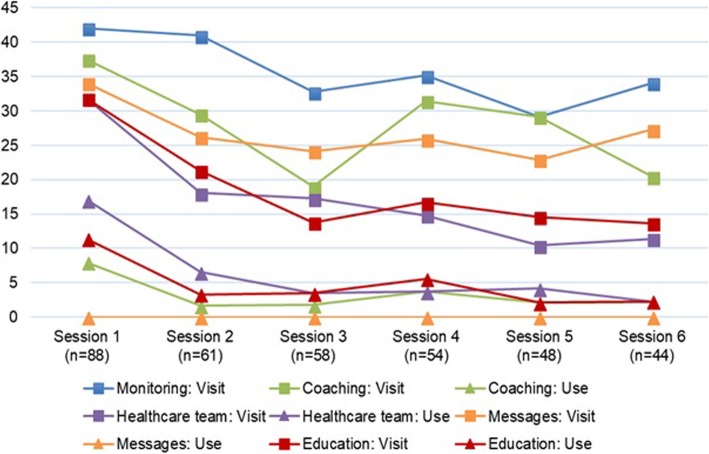
Usage per session and feature (COPD). Coaching action: adding a wish or challenge, starting or stopping a wish or challenge, adding a picture. Healthcare team action: select a caregiver, send a message. Messages action: sending a message. Education action: opening education topic. No specific actions could be specified for the monitoring data. % users = (number of different users visiting or using the feature in that session / total number of users in that session) * 100

In the Figs. 1, 2 and 3, the visits and the actual use of the different services of the PHRs are shown for T2DM, CHF, and COPD, respectively. The number of sessions on the x-axes of the figures are based on the mean number of logins per user of every PHR.

#### Number of sessions

After their first login, 181 of the 301 T2DM users (60%) returned for a second session and 50/301 users (17%) returned for six or more sessions. Half of all COPD users returned for six or more sessions. Since CHF patients were asked to perform their measurements on a daily basis, the results of the analyses show that the CHF PHR was used on a longer term. Therefore, the first 200 sessions are shown in Fig. 2. Ninety-one of 147 CHF users (62%) returned for a second session, 16/147 users (11%) returned for 101 or more sessions. Across the three PHRs, all services are viewed by the users, but the actual usage (e.g., adding measurements or goals) is lagging.

#### The services used

The education services were used the most. For the T2DM PHR, educational topics were opened by on average 20% of the users across the first six sessions, for the COPD PHR this percentage was 3%. Also, almost half of the CHF users (48%) actually opened the educational topics across the first 200 sessions. CHF patients used this education service together with the overviews of monitored values and target values, while COPD patients used the overview of healthcare providers as well. The coaching service was used the least by T2DM and COPD patients, CHF patients used the service for adding medication the least.

#### The use over time

In T2DM, the use of the education service increased after the fourth session, while the use of the monitoring service is slightly decreasing. In CHF, the percentage of individuals that use the overviews of monitored values is slightly increasing, indicating more targeted use when individuals return on the long term. The actual use of the education services is the least in sessions 41 to 50, but increases again after 50 sessions.

The use of the different services by the COPD patients is the highest in the first session, which was a training session. After the first session, the actual use of the different services decreases, but stays more or less constant over time. In comparison with the other PHRs, the service for education was relatively less used by the COPD users.

### Focus groups

The participants of the focus groups indicated that the services offered via a PHR should be interrelated. Services for coaching (see Table 1) are strongly associated with monitoring health values in order to gain insight into the progression towards the health-related goal) and education (what does the patient need to know to be able to reach a certain goal). At the same time, using services for monitoring and education are perceived as time-saving for caregivers as well, since monitoring data can be used during consultations, and patients can be directed towards educational topics on the PHR (instead of using consultations for education). Using valid and reliable tools is seen as an important prerequisite for performing the measurements at home.

Caregivers indicate that patients should contemplate about their health-related goals and add these to the PHR before a consultation, since this will support patients in organizing their thoughts, which can in turn support self-management. If necessary, the caregiver can support the patient in formulating achievable goals during a consultation. These goals should be leading in the treatment to keep patients motivated. To avoid disappointments, clear agreements should be made between patients and caregivers about if and when feedback is given on the progress of a patient. Based on the results of the focus groups, an example of a care pathway with the integration of a PHR was created. This pathway is included as an additional file (see Additional file 1).

### Interview study

Although all participating caregivers in the interview study were mainly enthusiastic regarding the use of a PHR in chronic care in order to support self-management skills of their patients, there were still some issues that hindered a successful implementation of the PHR in daily care routines (Table 3).

**Table 3:** Topics influencing the implementation of the PHR, according to care providers, including examples

Topic	Example
Training and guidance	‘Yes, that was not really difficult. The PHR, seeing health values, it is not very difficult.’
	‘… No idea how I had to work, so we sat with the manual next to the patient, we went through it step by step. But then the patient also had enough of it, no matter how positively you started it, at some point you notice your own frustration.’
	‘It was still vague what exactly we had to do and what was expected from us.’
	‘Well, after training you still need to try it yourself and look at the platform and the PowerPoint again. You need to show initiative by yourself, otherwise it is difficult.’
Compatibility with other systems	‘At this time, we work with three different systems: the PHR, our own EHR, and the telemonitoring system. We constantly need to switch between these three systems, that is just difficult. It is just not workable that way.’
	‘It should be user-friendly. You should not have four systems in which you have to fill the same information.’
	‘We must keep track of two systems, which is time consuming, it is not possible to have a link between the PHR and the EHR at the same time’
Integration in daily care routines	‘I must honestly say that we forget to look at the PHR. That is also because it is not in your daily routine.’
	‘I don’t feel like going to look there every day, but maybe once every month to take a look at what I can improve and how.
Use by patients	‘They are often also excited but in practice they still do not use it.’
	‘Because he did not fill in the medication and co-morbidities, while we clearly stated during inclusion, that they had to fill in so we could take it into account when health values deviate. The first person did not do it, the second neither, then you fall back on your own system.’
Usability	‘There were a lot of things in this platform, that did not work. In addition, it was not really an enchanting site and especially in this hospital very slow. We did not really work with the PHR, I must say.’
	‘You can see recent values, and the last 2 weeks. That is also a limitation, because sometimes you want to see for a longer term.’
Communication via PHR	‘Implement a certain learning element. For example, that the system indicates: ‘You have now gained two pounds, do you think that the care giver should take action or are you capable to do something on your own?’
	‘For example, a digital contact with the patient. Especially with younger patients, or people who are used to e-mail. Contact them via this way.’

#### Training and guidance

All participating nurses (in all three groups) were trained once or twice in the services the PHRs offer and the protocols for the evaluation studies. After these training sessions, the nurses mostly felt that the PHRs were easy to use, but they did not feel they had a comprehensive grasp on how to integrate these into their daily working routines.

#### Compatibility with other systems

An important and frequently raised issue among all participating caregivers, was the incompatibility of the PHR with the other systems the caregivers had to work with. All caregivers are using at least an EHR and an email client in their daily working routines, between which they often had to switch. Adding a new system to this routine was perceived as impractical and labor-intensive. The caregivers in the CHF project for example, were asked to check the monitored health values of their patients on a daily basis in the telemonitoring system. When values of a patient were deviating, the HF nurses first needed to access the PHR to gain insight into changes in medication and/or co-morbidities. Subsequently, the patient had to be contacted, where every follow-up action needs to be registered in the EHR as well.

#### Integration into daily routines

A third barrier in the deployment of the PHRs was the lack of integration within daily care routines. Some caregivers indicated that only a very small proportion of their patient population is an (active) user. As a result, the services of the PHRs hardly played a role during consultations with the patients. According to the participants, this had different consequences, such as forgetting to mention the PHR during consultations, and difficulties with establishing new working routines. In the CHF project for example, the medication overviews could serve as an extra information source for caregivers when deviating telemonitoring values are being signaled. However, the caregivers experienced that these overviews are often not accurate, not kept up-to-date, or not filled in at all. Therefore, HF nurses indicated that it is more efficient to directly call the patient when values are deviating, instead of checking the overviews first.

Using the PHR was therefore perceived as time-consuming instead of supportive. However, care providers do expect that, when the PHR is better integrated, workload will reduce and patients will be stimulated to take their own responsibilities which will in turn support self-management behavior.

#### Usability

The usability of the PHRs and the visualization of data for caregivers are seen as important barriers for using the PHR as well. The PHR is perceived as slow and unclear, and instructions were sometimes perceived as patronizing (according to COPD caregivers). Furthermore, patients and caregivers often did not receive their login credentials or logging in was not possible due to technical issues.

#### Communication via the PHR

Caregivers in the three research groups indicated that they would value the opportunity for short interactions with their patients via the PHRs, for example via a chat or messaging function. They noted that patients are sometimes left with unanswered questions, for example when they see deviating health values. Users should then be able to find an answer via the PHR to the first questions they may have (e.g., are values indeed deviating, and why could that be? What should I do now?). Furthermore, HF nurses indicate that they often ask the same questions to their patients in case telemonitoring values are deviating. A short questionnaire comprising health-related questions for patients to complete after every measurement would increase the efficiency of the telemonitoring system and help the HF nurses to increase the accuracy of the decisions that are made based on the telemonitoring values. Thus, improving the interaction with the PHR will eventually improve the communication between patients and caregivers.

#### Perceived usability among potential end-users

The usability tests among three potential T2DM end-users showed that they perceive the PHR as appealing and inviting at first sight. However, they did expect a welcome message and a short explanation regarding the goal and the features of the PHR. As soon as the potential end-users were invited to execute the scenarios, it became clear that the structure of the system did not match their expectations. For example, they expected a function for monitoring blood glucose levels (which was not offered), and within the coaching feature, the concepts of ‘wishes’, ‘goals’ and ‘actions’ remained unclear. Furthermore, participants found it confusing that some elements (e.g., videos and some information topics) were opened in a new window.

The 10 usability tests among potential end-users of the COPD PHR revealed that participants experienced difficulties with finding the right information. Because there are many buttons in one screen, the lay-out was perceived as unclear. Some elements at the home-page look like buttons but are not, which caused confusion. Furthermore, the coaching function is perceived as unclear. For the participants, the distinction between different concepts (e.g., wish, challenge) is unclear, as well as how to add wishes, action plans, and evaluations to the PHR.

The participants in both the T2DM and COPD groups indicate that they would like to use the PHR in the future (mainly to use the monitoring function), given that the usability will be improved and that the caregivers will use it in daily practice as well. In that case, participants would value a replacement of regular consultations as well.

## Discussion

The aim of this study was to evaluate the fidelity of a PHR for patients with type 2 diabetes mellitus (T2DM) congestive heart failure (CHF), or COPD and how it influenced the effectiveness. By using a convergent parallel mixed-methods design, we gained a more complete understanding of how caregiver responsiveness and the quality of delivery of the PHR influenced the actual use of the PHR by patients; to what extent this use matched the use that adds value to the working routines in primary and secondary care, according to caregivers (the intended use or differentiation), and how any differences between the actual and intended use could be explained.

The results showed that with the development and implementation of the PHR, the main problem was identified was on the level of the delivery of the PHR: A technology was offered to patients and their caregivers, without their involvement during earlier phases of development and without any guidance regarding the integration of the PHR into the daily working routines of caregivers. The PHR was mainly introduced in the light of the evaluation study and the training of the caregivers predominantly focused on how to collect the data for this study. As a result, caregivers did not know what was expected from them with regards to using the services of PHR, and were unaware on how to deploy these in daily care routines. Because the workload is perceived as high, caregivers experienced that they had insufficient time to explore what the PHR could potentially mean for their working routines and as a result, the PHR was seen as a burden on top of the regular care. Therefore, caregivers find it difficult to motivate their patients in using the PHR, and the log data analyses showed that the actual use (the dose) of the PHR by patients is lagging as well. This limited use is common in PHR evaluations [37] and several recent systematic reviews focusing on the implementation of complex (eHealth) interventions and PHRs stress that the (perceived) fit of eHealth technologies with the current working routines and the interoperability with other systems are key factors for a successful implementation [16, 37, 38]. In turn, caregiver responsiveness might be the key to patient responsiveness [20, 39–41].

**Fig. 4:**
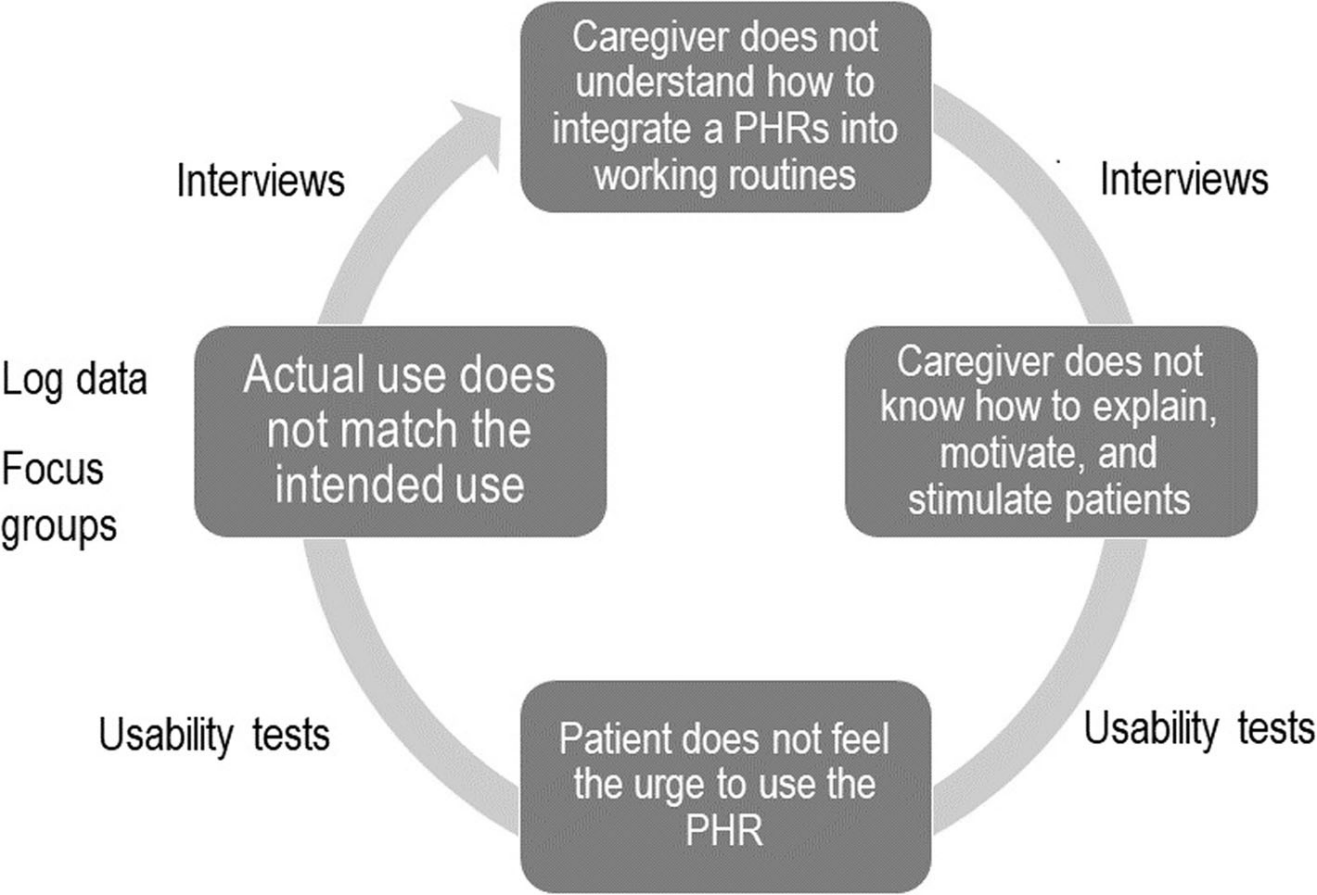
Synthesis of the results, with an indication of the data sources that were used to identify every step

Moreover, it is likely that the usability of the PHR influenced its actual use as well. Many T2DM caregivers indicated prolonged troubles with logging in and participants of the usability tests perceived the designation of the concepts within the coaching service (e.g., *Wish*, *Goal*, and *Action*) as unclear and uninviting to work with. This finding might explain why the coaching service (which was perceived as an essential feature to reach the intended effects during the focus group meetings) was used by so few users. Figure 4 depicts an overview of this vicious circle, with an indication of the data sources that were used to identify every step.

### Implications and recommendations

The results of this study show that a holistic approach of eHealth development, implementation, and evaluation is crucial in the success of the project. eHealth is not just a tool which will be implemented automatically once it is offered to the end-users without considering their needs, wishes, and concerns. Rather, considering the whole of the interactions between the system, the user, and the context in which the eHealth technology is deployed is of utmost importance to understand the fidelity of a technology. These results are consistent with previous systematic reviews focusing on eHealth implementation, stressing that the (perceived) fit of eHealth technologies with the current working routines and the interoperability with other systems were key factors for a successful implementation [16, 38]. Furthermore, the findings of the current studies are supported by Nazi, who emphasizes the role of the caregivers in the adoption of PHRs by the patients [18]. Therefore, decent training and support of all end-users in deploying the system is necessary for the quality of delivery, and creating engagement, but when the first impression is modest and the technology does not fit the wishes, needs, and the context of end-users (and user responsiveness is thus lacking), it will hardly or not be used.

In this view, considering the fidelity of technology such as PHRs is not a post-design step, and the conditions for a successful implementation should be taken into account already in early phases of eHealth development [42]. It is thus of utmost importance to involve all stakeholders, including the end-users, already in early stages of development to gain insight into their needs and wishes. However, the inquiry of the context is just as important [19, 42], for example by exploring national guidelines or by observing the daily (working) routines of caregivers and patients. Short and iterative development cycles addressing the technology and its interaction with the users and the context are crucial [38], because it allows for problems to be identified already early in the development, increasing the chances for a successful implementation [19, 42].

To be able to understand what differences a technology can make and why some technologies are effective whilst others are not, data from differences sources in a mixed-methods approach has proven to be of added value [22]. In this study, focusing on (the relations between) multiple concepts related to intervention fidelity provided explanations for the disappointing results of e-Vita so far, that could not be revealed by solely focusing on the effectiveness of the technology in an experimental trial. In this research, the log data had the potential to provide objective insights into the actual usage of the different services of e-Vita, but could only be put into perspective by using the results from the focus group study, interview studies and usability tests [33]. At the same time, only focusing on the experiences of caregivers would not have given insight into the influence on the actual usage of the PHR. Thus, understanding the interplay between the technology, its users and the context of implementation are of utmost importance when understanding the effectiveness.

### Limitations

An important limitation of this research was that during the focus groups, only insights from T2DM caregivers could be obtained. This might have decreased the generalizability of the results with respect to the other disease groups. However, because we focused on the aspects that are similar between disease groups and used a mixed methods approach, we could extrapolate these finding to CHF and COPD.

Furthermore, because of the large number of participants across the three trials, we were able to interview only a small group of a diverse population. However, despite this variation, no new insights were obtained from the last conducted interviews and we therefore feel that saturation has been reached. Furthermore, by combining the interview data with objective log data from all users in the mixed-methods approach, we were able to cope with this potential limitation.

## Conclusions

The results of this study showed that the lacking effects of a PHR for patients with chronic diseases can be explained by assessing the fidelity. The results revealed that, the actual use of the PHRs by the patients (the dose) is strongly influenced by the responsiveness of caregivers. In turn, the responsiveness is likely to be strongly influenced by the perceived support to formulate the needs regarding the differentiation and delivery of the PHR. It could thus be concluded from the results of this study that it is of utmost importance to involve all end-users already in early stages of eHealth development to increase the added value and to facilitate the implementation. Short and iterative development cycles are crucial for identifying problems already in early stages of development and to increase the chances for a successful implementation. When evaluating fidelity, a mixed methods approach is of added value in providing explanations for the found effects that could not be revealed by solely focusing on the effectiveness of the technology in an experimental trial.

## Supplementary information

**Additional file 1.** An example of a care pathway with the integration of a PHR for T2DM after the diagnosis.

## Data Availability

The datasets used and/or analyzed during this study are available from the corresponding author on reasonable request.

## Abbreviations

CHF: Congestive heart failure
COPD: Chronic Obstructive Pulmonary Disease
PHR: Personal Health Record
T2DM: Type 2 diabetes mellitus
