# Supplementary figures and images for: Evaluating the implementation of a personal health record for chronic primary and secondary care: a mixed methods approach
Source: BMC Med Inform Decis Mak. 2019 Nov 27;19:241. doi: 10.1186/s12911-019-0969-7 (PMC6882368; doi:10.1186/s12911-019-0969-7)

## Appendix 1. An example of a care pathway with the integration of a PHR for T2DM after the diagnosis

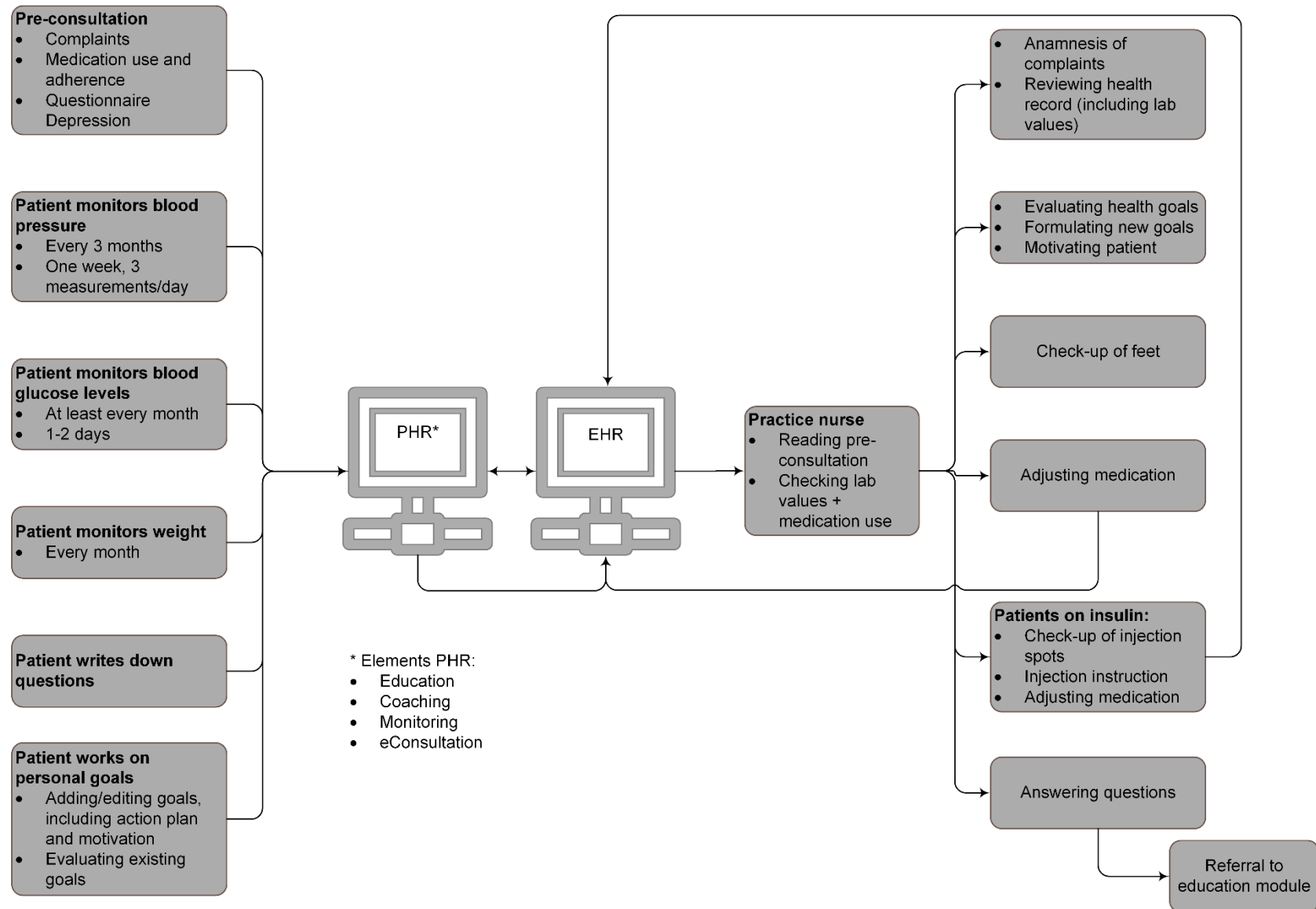

Supplement: Supplementary file 1 — Additional file 1. An example of a care pathway with the integration of a PHR for T2DM after the diagnosis. [file 12911_2019_969_MOESM1_ESM.pdf]
